# Assessment of proprioceptive decline in chronic ankle instability: a comparative evaluation of sub-modalities

**DOI:** 10.3389/fspor.2025.1717681

**Published:** 2026-01-12

**Authors:** Junjun Li, Qi Wang, Xinmeng Zhang, Yujing Cao, Wenqi Ran, Qipeng Song

**Affiliations:** 1 College of Sports and Health, Shandong Sport University, Jinan, China; 2 Sport Science School, Beijing Sport University, Beijing, China; 3 College of Physical Education, Shandong Normal University, Jinan, China

**Keywords:** central detection, pre-activation, proprioceptors, recurrent ankle sprains, thixotropy

## Abstract

**Purpose:**

Recurrent ankle sprains in chronic ankle instability (CAI) individuals are strongly linked to impaired proprioception, but the specific mechanisms underlying these impairments remain incompletely understood. This is largely due to the complexity of the proprioceptive system, which comprises multiple sub-modalities such as kinesthesia, joint position sense, force sense, vibration sense and landing proprioception, with the characteristic deficits of these sub-modalities and their diagnostic sensitivity and specificity remaining unclear. This study aims to comprehensively assess proprioception in CAI individuals by pointing sub-modality deficits, and to identify the core sub-modality with the greatest clinical discriminative value for CAI.

**Methods:**

Fifty-eight participants were recruited, including 29 with CAI and 29 healthy controls. They underwent a battery of proprioceptive assessments: kinesthesia via the Threshold to Detect Passive Movement (TTDPM) test, joint position sense through the Joint Position Reproduction (JPR) test, force sense with the Force Match (FM) test, vibration sense using the Vibration Detection Threshold test, and landing proprioception evaluated by the Ankle Inversion Discrimination Apparatus for Landing (AIDAL) test. Between-group differences were analyzed using independent samples *t*-tests, Mann–Whitney *U* tests, and AUC tests.

**Result:**

Higher passive perception thresholds in TTDPM tests (dorsiflexion: *p* = 0.03; inversion: *p* = 0.004; eversion: *p* = 0.018), higher absolute errors in FM tests (plantarflexion: *p* = 0.025; dorsiflexion: *p* = 0.043; inversion: *p* = 0.018; eversion: *p* = 0.014) and lower AIDAL score (*p* = 0.002) were detected in people with CAI. ROC curve analysis of the tests with significant intergroup differences revealed that the AIDAL exhibited the highest discriminative ability (AUC = 0.728).

**Conclusion:**

Proprioceptive deficits in CAI are modality-specific, primarily affecting kinesthesia, force sense, and landing proprioception. Among the impaired sub-modalities, landing proprioception has the strongest predictive value for CAI and can serve as a key target for clinical assessment and rehabilitation intervention.

## 1 Introduction

Ankle sprains are among the most prevalent sports injuries, accounting for 16%–40% of all sports injuries (1). In the United States, roughly 825,718 people sustain acute ankle sprains annually (2), incurring medical costs of about $6.2 billion (3). Up to 70% of these people may experience recurrent sprains, “giving way” episodes, ankle instability, and potentially develop chronic ankle instability (CAI) (3).

Proprioceptive deficits have been identified as a core risk factor for recurrent ankle sprain in people with CAI (4). In the updated model of CAI proposed by Hertel and Corbett (5), sensory perception and motor behavior impairments were established as core components, which has sparked extensive research and discussion. A large number of studies have confirmed that the “reduced somatosensory perception” caused by the damage to mechanoreceptors due to the initial ankle sprain is a key mechanism for the recurrent ankle sprain in people with CAI (6). Specifically, an initial lateral ankle sprain may impair the proprioceptors, leading to delayed or disrupted sensory signals. This results in the failure to initiate protective ankle eversion in time to correct excessive inversion, consequently increasing the risk of recurrent sprain (7).

Proprioception is defined as a multidimensional perceptual system that determines the position and movement of body segments in space (8). In practice, it can be classified into distinct sub-modalities based on task demands, each associated with unique information-processing mechanisms and functional implications (9). Specifically, proprioception encompasses several task-oriented sub-modalities: kinaesthesia, which discriminates the amplitude and velocity of fine joint movements; joint position sense, which perceives the static position of a joint; force sense, which detects changes in limb load and muscle tension; vibration sense, which is sensitive to mechanical vibratory stimuli; and landing proprioception, which identifies ankle joint angles or subtle movements during dynamic tasks (10, 11).

Given that proprioception is widely recognized as a multimodal system, it is logically plausible to deduce that specific sensory integration pathway may sustain damage from prior ankle sprains in people with CAI, consequently resulting in deficits within certain proprioceptive sub-modalities. Despite extensive research conducted on various proprioceptive sub-modalities, the question of which sub-modalities are impaired in CAI people remains a subject of debate, with conflicting findings reported. For instance, while some systematic reviews (12) indicate that people with CAI exhibit significantly poorer joint position sense and kinesthesia compared to healthy control (HC) people, others (13) report no statistically significant differences. Xiao et al. (14) confirmed clear force sense deficits in CAI people, whereas lee et al. (15) suggested such impairments are only evident in specific movement directions. Furthermore, research on the performance of landing proprioception and vibration sense in CAI people remains limited, and it is still unclear whether these sensory modalities are impaired in people with CAI. Identifying the characteristics of proprioceptive deficits in the CAI population may enhance our understanding of the underlying mechanisms contributing to proprioceptive decline in this population.

More importantly, currently available research has not yet analyzed the predictive value of various sub-modalities for individuals with CAI. This gap makes it impossible to answer a fundamental clinical question: among the various proprioceptive sub-modalities, which are the most sensitive and specific indicators for CAI? Clarifying this question is crucial for enabling precise assessment and targeted rehabilitation.

The objective of this study is to comprehensively evaluate proprioception in individuals with CAI, by identifying deficits in proprioceptive sub-modalities, and to further employ ROC curve analysis to determine the core indicator with the greatest clinical discriminative value for CAI from among the impaired sub-modalities. It is hypothesized that people with CAI have worse kinesthesia, joint position sense, force sense, vibration sense, and the landing proprioception, and that the discriminative power of these sub-modalities for identifying CAI would be comparable.

## 2 Materials and methods

### 2.1 Sample size estimate

An *a priori* sample size calculation was performed using G*Power software (Version 3.1), with the Type I error set at 0.05 (*α* = 0.05) and statistical power at 0.80. Based on previously reported effect sizes (TTDPM: CAI: 3.01 ± 2.01, HC: 1.7 ± 0.80, *d* = 0.85 (16, 17); JPR: CAI: 3.07 ± 1.33, HC: 2.15 ± 1.04, *d* = 0.77 (18); FM: CAI: 0.049 ± 0.03, HC: 0.031 ± 0.02, *d* = 0.75 (18); AIDAL: CAI: 0.777 ± 0.05, HC: 0.815 ± 0.05, *d* = 0.76 (19)), the calculated minimal sample sizes were 23, 27, 19, and 15 participants per group, respectively. To satisfy statistical power requirements across all four test modalities (prioritizing the maximum group size of 27), a final cohort of 29 participants per group was enrolled.

### 2.2 Participants

Participants were recruited from a local university through the distribution of flyers and delivery of presentations, yielding 76 people willing to participate in the study. Based on the international ankle consortium guidelines (20), the inclusion criteria for CAI participants were: (a) a history of at least one severe ankle sprain within 12 months prior to the study, resulting in pain, swelling, and restricted mobility for ≥1 day; (b) age between 18 and 25 years; (c) ≥2 recurrent sprain episodes in the past 6 months; (d) persistent ankle instability and dysfunction during daily activities; and (e) a Cumberland Ankle Instability Tool (CAIT) score ≤24. Exclusion criteria for CAI participants included: (a) history of lower limb fractures or surgeries; (b) acute lower limb injuries (e.g., sprains) within the preceding 3 months; (c) neurological disorders, diabetes, or vestibular diseases; and (d) history of bilateral ankle sprains, injuries, or bilateral chronic ankle instability. Inclusion criteria for HC were: (a) no history of ankle sprain, injury, or instability; (b) CAIT score ≥28; and (c) age between 18 and 25 years. The exclusion criteria for HC are the same as above. 58 participants were enrolled, including 29 with chronic ankle instability (female = 9, male = 20; age: 20.45 ± 1.80 years; height: 1.74 ± 0.09 m; weight: 67.69 ± 11.9 kg; CAIT score: 17 ± 2.35) and 29 without CAI (female=13, male=16; age: 20.96 ± 1.84 years; height: 1.71 ± 0.65 m; weight: 67.59 ± 12.30 kg; CAIT score: 27.72 ± 1.89). Human participation was approved by Institutional Review Boards in Shandong Sport University (2025) and was in accordance with the Declaration of Helsinki, and all participants provided written informed consent prior to data collection.

### 2.3 Threshold to detect passive movement (TTDPM) test

**Figure 1:**
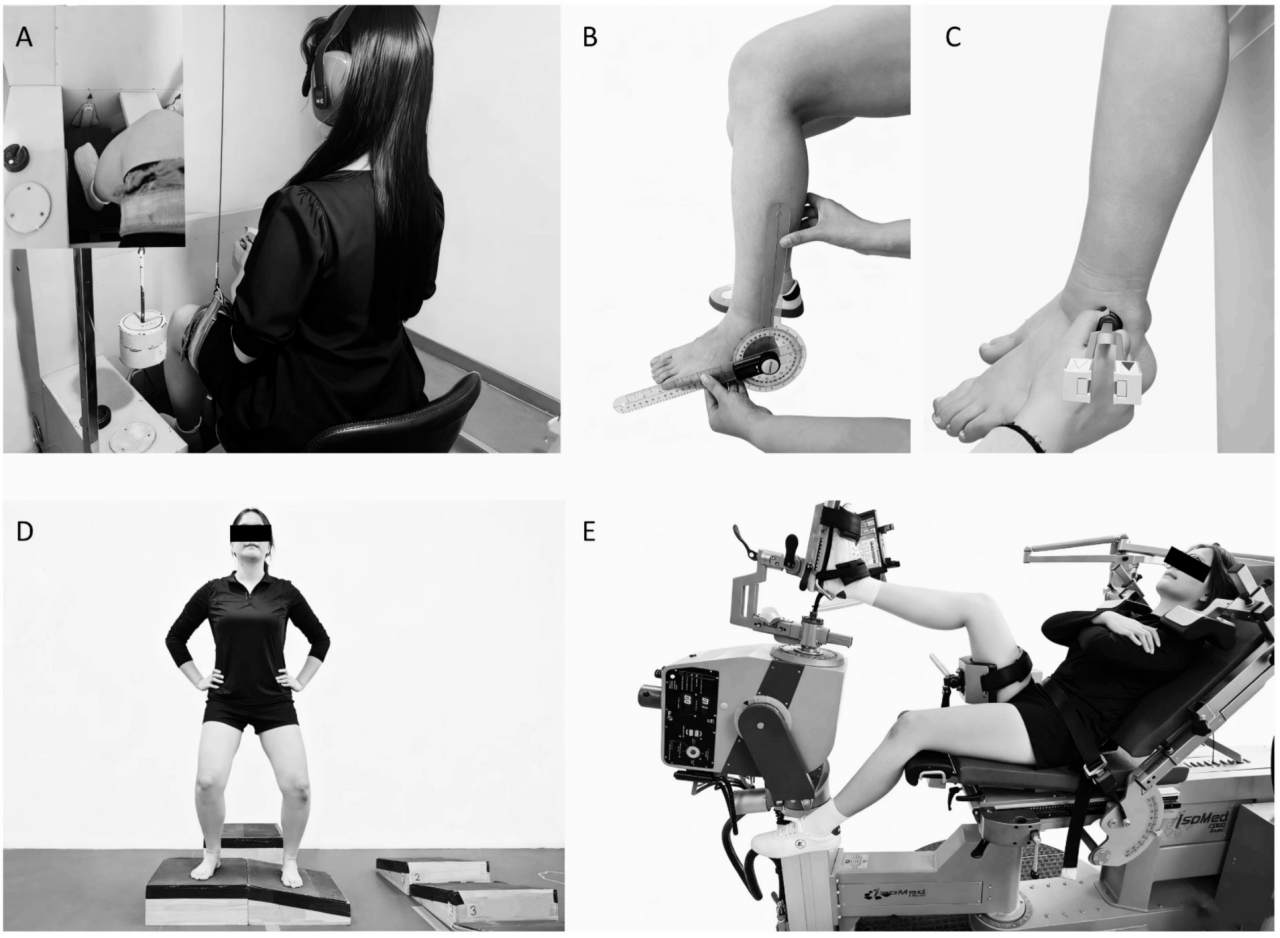
Test illustrations. **(A)** Threshold to detect passive movement test, **(B)** joint position reproduction test, **(C)** vibration disappearance threshold test **(D)** ankle inversion discrimination apparatus for landing test, and **(E)** force match test.

Kinesthesia in the directions of plantarflexion, dorsiflexion, inversion and eversion of the ankle joint were evaluated using specialized proprioceptive testing equipment (Tosimi Company, Jinan, Shandong, China) (21). When the examiner pressed the start button, the platform rotated at a constant angular velocity of 0.4 °/s. Participants were instructed to depress a handheld switch upon detecting movement, and tell the testers the direction of movement (e.g., inversion or eversion); the platform angular displacement at that moment was recorded as the kinesthesia (22) (Figure 1A). To diminish cutaneous cues, approximately 50% of the lower-limb weight was borne by the platform and a thigh-cuff suspension system limited further foot–platform contact. Participants wore blindfolds and noise-cancelling headphones to exclude visual and auditory input. All participants completed three valid trials, defined as the trial the movement direction was correctly identified, with a 30-second interval between trials.

### 2.4 Joint position reproduction (JPR) test

The JPR test was conducted using an electronic protractor (CemRed, Guilin, China) to assess joint position sense (18). Participants sat on a 60-cm-high platform with the lower limbs hanging naturally. During the initial phase of the test, the examiner used an electronic goniometer to adjust the ankle joint to the target angle (30 ° plantarflexion (23), 15 ° dorsiflexion (18), 20 ° inversion (23), and 15 ° eversion (18)), maintaining this position for 5 s (18). Subsequently, participants were instructed to actively reproduce the target angles without receiving any visual or verbal feedback. (Figure 1B) The absolute error (AE), defined as the discrepancy between the target and reproduced angle, was calculated for each trial. All participants completed three trials, with a 15-second interval between trials.

### 2.5 Force match (FM) test

The FM test was conducted using an ISOMED 2000 isokinetic dynamometer (D&R GmbH, Germany) to assess force sense. Initially, participants performed three maximum voluntary contractions (MVC) trials to determine peak torque. The values obtained were used to calculate 25% MVC, which served as the target intensity for the subsequent force-matching task. During the task, participants were asked to maintain the target torque as stably as possible for 5 s while viewing the real-time torque curve on the screen. Subsequently, the display was turned off, and participants were instructed to reproduce the target torque from memory. They indicated when they believed the target had been reached; torque was recorded for the following 5 s (24) (Figure 1E). The AE values the target torque (set at 25% MVC) and the reproduced torque were calculated to evaluate performance. AE values were normalized to body weight to control for inter-individual differences in force perception. All participants completed three valid trials, defined as trials devoid of significant torque fluctuations during the 5-second target torque generation phase, with a 15-second interval between trials.

### 2.6 Vibration disappearance threshold (VDT) test

Vibration perception was evaluated with a 128-Hz Rydel-Seiffer tuning fork (graded 0–8; Gimmer GmbH, Germany; mass: 120 g) by recording the minimum vibration disappearance time threshold at medial malleolus, lateral malleolus, and first metatarsal head (25). Participants sat on a height-adjustable chair with their lower limbs relaxed. The examiner strikes the tuning fork against the metacarpal bone of their hand. When the amplitude indicator reaches the 6.5 mark, the fork was promptly applied to the predetermined bony landmarks with consistent pressure (approximately 5 N) to ensure reliable vibration transmission. A stopwatch was started simultaneously and given to the participant, who was instructed to press it the instant the vibration ceased (25) (Figure 1C). All participants completed three trials, with a 30-second interval between trials.

### 2.7 Ankle inversion discrimination apparatus for landing (AIDAL) test

The AIDAL test was conducted using the AIDAL system (19) to assess landing proprioception. Participants stood barefoot on a starting platform, hands on the iliac crests, head facing forward and eyes looking straight ahead. They were instructed to extend the test limb forward and drop-land so that the tested foot contacted a wedge-shaped platform while the contralateral supporting foot landed on a horizontal surface. During familiarization, participants completed 12 trials (3 repetitions per inversion angle) in ascending order to learn the spatial configurations of the platforms (Figure 1D). In the formal phase, wedge angles were randomized to prevent anticipatory responses. Immediately after landing, participants verbally reported the perceived inversion angle (1–4, corresponding to 10 °–16 °) without feedback. A total of 40 consecutive trials were conducted (26), correct and incorrect responses were recorded for each angle to quantify proprioceptive accuracy.

### 2.8 Data reduction

For TTDPM, JPR, VDT and FM tests, the mean of three trials was used in subsequent analyses. In the AIDAL test, after collecting the response data of all participants in each trial, the frequency of the selected numbers chosen by each participant at each slope was first organized into a 4 × 4 matrix. Subsequently, the paired comparison of the cumulative frequency of their responses (1–2, 2–3, 3–4) was conducted using the Receiver Operating Characteristic (ROC) analysis, and the area under the curve (AUC) is calculated as the AIDAL score, representing the sensitivity of the landing proprioception (19).

### 2.9 Data analysis

Data analysis was conducted using SPSS (version 27.0, IBM). All variables were described as mean ± standard deviation (Mean ± SD). The Shapiro–Wilk test was performed to assess data distribution normality. Independent *t*-tests (for normally distributed data) or Mann–Whitney *U* tests (for non-normal distributions) were employed to compare proprioceptive thresholds between people with CAI and HC using five assessment methods. Gender is analyzed using the chi-square test. Effect sizes for group differences were evaluated using Cohen's d (parametric data) or *η*² (non-parametric data). The thresholds for Cohen's d were as follows: <0.20, trivial; 0.21–0.50, small; 0.51–0.80, medium and >0.81, large. The thresholds for *η*² were as follows: >0.01, small; >0.06, medium; and >0.083, large (27, 28). Moreover, whenever a statistically significant between-group difference was observed among the proprioceptive tests, a diagnostic analysis using ROC curves was conducted to evaluate their discriminative capacities based on the AUC. An AUC value of 0.5 indicates that the response is at a random level, and 1.0 indicates complete discrimination (16, 17).

## 3 Results

The Shapiro–Wilk tests indicated that the plantarflexion, dorsiflexion, and inversion angles obtained from the TTDPM test, along with the plantarflexion and eversion torques measured in the FM tests were non-normally distributed, and their between-group differences were compared using Mann–Whitney *U* tests; other variables were normally-distributed, and they were compared using independent sample *t*-tests. The baseline characteristics of all participants are presented in Table 1, with no significant differences observed in any items except for the CAIT score.

**Table 1:** The baseline of the participants’ information.

Items	CAI (*n* = 29)	HC (*n* = 29)	*p*
Sex	9F, 20M	13F, 16M	0.279
Age (years)	20.45 ± 1.80	20.96 ± 1.84	0.297*
Height (m)	1.74 ± 0.09	1.71 ± 0.07	0.192
Weight (kg)	67.69 ± 11.9	67.59 ± 12.30	0.974
CAIT (scores)	17 ± 2.35	27.72 ± 1.89	**0**.**001**

CAI, chronic ankle instability; HC, healthy controls; CAIT, cumberland ankle instability tool.

* Analyzed by Mann–Whitney *U* tests, others by independent sample *t*-test.

The descriptive statistics for all variables, including means, standard deviations, *p*-values, and effect sizes for TTDPM, JPR, FM, VDT and AIDAL tests, are presented in Table 2. Higher passive perception angles in ankle dorsiflexion (*p* = 0.030, *η*^2^ = 0.099), inversion (*p* = 0.004, *η*^2^ = 0.104) and eversion (*p* = 0.018, *d* = 0.645) in TTDPM tests; higher absolute angular error in ankle plantarflexion (*p* = 0.025, *η*^2^ = 0.063), dorsiflexion (*p* = 0.043, *d* = 0.565), inversion (*p* = 0.018, d = 0.723), and eversion (*p* = 0.014, *η*^2^ = 0.106) in FM tests, and lower AIDAL score (*p* = 0.002, *d* = 0.087) were detected in people with CAI. No significant differences were observed in TTDPM (plantarflexion: *p* = 0.258), JPR (plantarflexion: *p* = 0.109; dorsiflexion: *p* = 0.055; inversion: *p* = 0.051; eversion: *p* = 0.069), and VDT (Medial malleolus: *p* = 0.067; Lateral malleolus: *p* = 0.079; 1st metatarsal head: *p* = 0.086) tests.

**Table 2:** Descriptive characteristics of the outcome variables.

Tests	Variables	CAI	HC	*P*	*d*	*η* ^2^
TTDPM	Plantarflexion	1.02 ± 0.57	0.86 ± 1.59	0.258*	–	0.005
	Dorsiflexion	1.30 ± 0.67	0.95 ± 0.33	**0**.03*	–	0.099
	Inversion	3.3 ± 1.90	2.09 ± 1.64	**0**.004*	–	0.104
	Eversion	3.27 ± 1.75	2.35 ± 1.00	**0**.**018**	0.645	–
JPR	Plantarflexion	3.67 ± 1.12	3.21 ± 1.01	0.109	0.431	–
	Dorsiflexion	1.96 ± 1.15	1.50 ± 0.59	0.055	0.503	–
	Inversion	2.88 ± 0.95	2.41 ± 0.87	0.051	0.516	–
	Eversion	2.95 ± 0.58	2.67 ± 0.54	0.069	0.499	–
FM	Plantarflexion	0.09 ± 0.039	0.07 ± 0.038	**0**.025*	–	0.063
	Dorsiflexion	0.083 ± 0.03	0.070 ± 0.03	**0**.**043**	0.565	–
	Inversion	0.068 ± 0.03	0.051 ± 0.02	**0**.**018**	0.667	-
	Eversion	0.03 ± 0.014	0.02 ± 0.015	**0**.014*	–	0.106
VDT	Medial malleolus	9 ± 1.66	9.83 ± 1.69	0.067	0.496	–
	Lateral malleolus	8.91 ± 1.85	9.67 ± 1.35	0.079	0.469	–
	1st metatarsal head	10.09 ± 2.44	11.04 ± 2.44	0.086	0.389	–
AIDAL	AIDAL score	0.73 ± 0.042	0.77 ± 0.045	**0**.**002**	0.087	–

CAI, chronic ankle instability; HC, healthy controls; TTDPM, threshold to detect passive motion; JPR, joint position reproduction; FM, force match test VDT, vibration disappearance threshold test; AIDAL, the ankle inversion discrimination apparatus for landing. Bolded numerical values indicate statistically significant differences.

* Analyzed by Mann–Whitney *U* tests, others by independent sample *t*-test.

**Figure 2:**
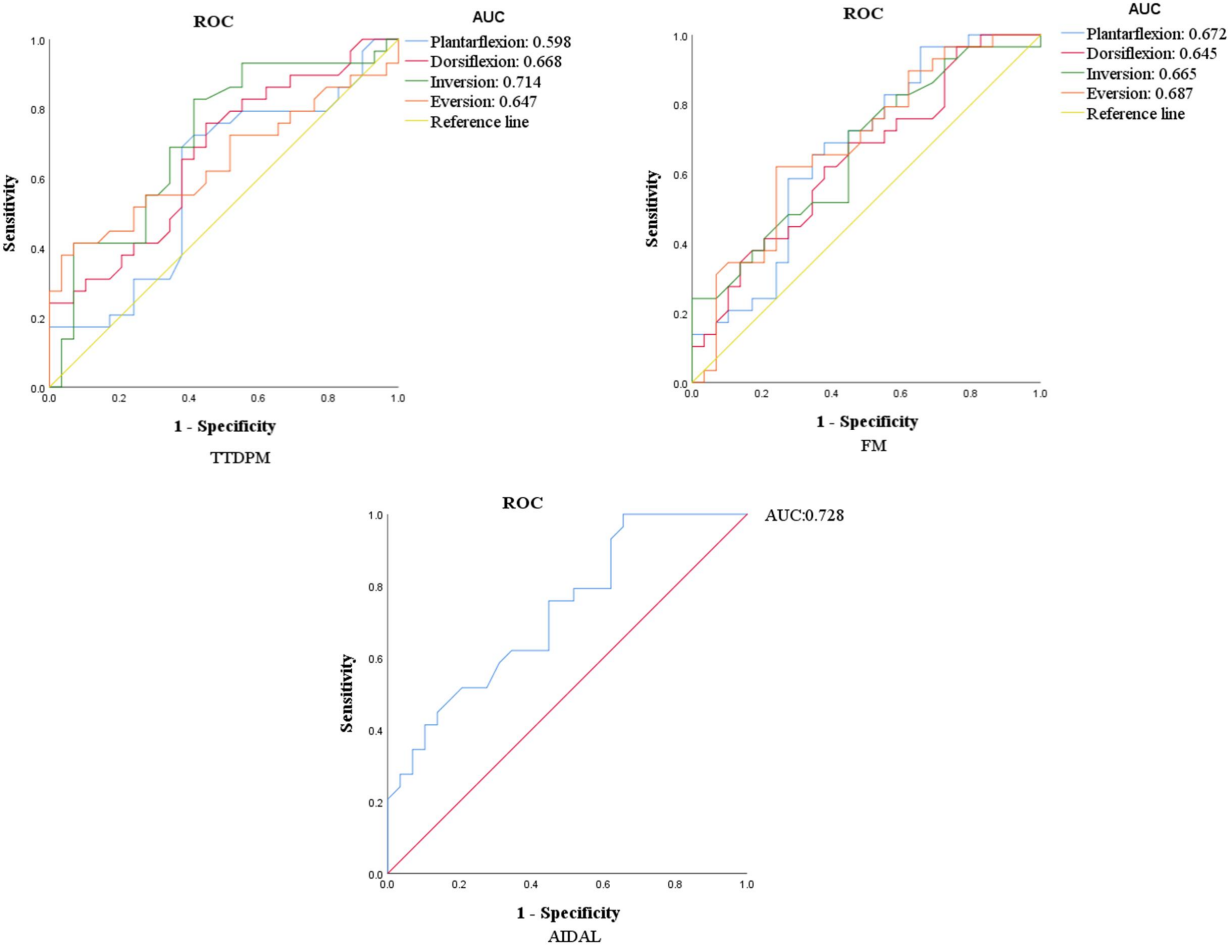
ROC curves of proprioceptive sub-modalities. TTDPM, threshold to detect passive movement test; FM, force match test; AIDAL, ankle inversion discrimination apparatus for landing test; ROC, receiver operating curve; AUC, area under the curve.

The ROC curves representing the discriminatory efficacy for between-group differences are presented in Figure 2. The ROC analysis revealed that the AUC value (0.728) of AIDAL score was the highest. This was followed by the AUC value of the dorsiflexion (0.668), inversion (0.714), and eversion (0.647) angles in the TTDPM test, as well as the plantarflexion (0.672), dorsiflexion (0.645), inversion (0.665), and eversion (0.678) torque in the FM tests.

## 4 Discussion

This study is the first to comprehensively investigate the characteristics of proprioceptive sub-modalities in a CAI cohort. The results show that proprioceptive impairments in individuals with CAI are modality-specific, primarily manifested in the sub-modalities of kinesthesia, force sense, and landing proprioception. Moreover, the discriminative ability of different sub-modalities for CAI people is inconsistent, with landing proprioception showing higher discriminative power than kinesthesia and force sense. This suggests that this modality may be more strongly associated with the mechanism of recurrent ankle sprains in CAI people. The average values and ranges of the proprioceptive test results in this study are similar and comparable to those of previous studies using the same test methods (29–32).

Proprioceptive impairments in CAI people are heterogeneous and modality-specific. A common misconception in existing research is that results obtained with the use of one particular method can be generalized. This reflects an implicit assumption that a generalizable proprioceptive ability exists and that each test measures this general ability, overlooking the multimodality of proprioception. If this would be the case, a strong association between results obtained with different tests. However, our findings do not support the existence of such a strong correlation. De Jong et al. and Yang et al. (33, 34) found that no correlations existed between ankle joint position sense, movement sense, and movement discrimination sensitivity, reinforcing the concept of modality-specific deficits. This may occur because different sub-modalities rely on distinct peripheral and central pathways (35). An ankle sprain may disrupt only specific signal transmissions, leading to selective functional impairments based on task demands.

The specific proprioceptive deficits observed in CAI people be explained by a selective injury mechanism to peripheral receptors during ankle sprains. During a lateral ankle sprain, the soft tissues around the ankle are subjected to intense stretching beyond the normal physiological range (36). This mechanical stress is transmitted to deeper tissues, causing structural damage to receptors and afferent nerves, thereby leading to proprioceptive deficits (37). However, due to differences in the anatomical locations and structural characteristics of the peripheral structures associated with different proprioceptive modalities (8), their susceptibility to sprains varies.

Specifically, the lateral ankle skin, owing to its higher elasticity, can effectively dissipate and absorb mechanical stress through deformation during stretching (38). Thereby, subcutaneous receptors such as Meissner corpuscles may be less susceptible to injury than muscle spindles and Golgi tendon organs. Additionally, within muscle spindles, the primary nerve endings responsible for sensing dynamic length are concentrated in the central nuclear bag region (39). Due to stress concentration, this area may undergo the greatest deformation during stretching, making primary endings particularly susceptible to damage. In contrast, secondary nerve endings, which detect static muscle length, are predominantly distributed in a reticular arrangement at the polar ends of intrafusal muscle fibers (40). These are exposed to relatively less stress during the process, allowing partial functional preservation. This differential peripheral receptor injury may explain why CAI people often exhibit significant deficits in kinesthesia, force sense, and landing proprioception, while joint position sense and vibratory perception remain relatively preserved.

The selective impairment pattern may also stem from differences in neural encoding properties among different modalities (35). A growing body of evidence suggests that recurrent lateral ankle sprains impair the ability of mechanoreceptors to promptly transduce mechanical stimuli into electrical signals, leading to delayed afferent input and consequently interfering with the function of related sensory modalities (41). However, compared with other sub-modalities, joint position sense and vibration perception may rely less on rapid signal conduction and instead may prioritize sustained representation of stimulus intensity and spatiotemporal patterns (42, 43). The conduction velocities of their respective afferent nerves further substantiate this point: Group II and Aβ afferent nerves exhibit significantly slower conduction speeds compared to Group Ia and Ib afferent nerves (44). Therefore, even in the presence of delayed peripheral signaling, these modalities may still be able to convey spatial location and rhythmic vibration information to the central nervous system via stable intensity–time coding. This may partly explain why people with CAI often exhibit relatively preserved joint position and vibration sense alongside marked deficits in kinesthesia and force sense.

A comparative analysis of impaired proprioceptive sub-modalities found that while both kinesthesia and force sense could differentiate between individuals with CAI and HC, landing proprioception yielded the highest diagnostic accuracy. The discriminative AUC value obtained from our study was comparable to Han et al. original study (19). The predictive utility of landing proprioception seems to be superior to other sub-modalities presumably because its assessment method provides exceptional ecological and functional validity. First, it assesses the sensorimotor system under real-world, sport-specific condition during landing, rather than in an artificial, static laboratory setting (6). Second, it captures proprioceptive acuity at the critical, high-risk moment of initial ground contact (0–120 ms), precisely when ankle sprains occur and rapid detection is essential for injury prevention (45). Third, its design aligns with modern motor control theory, which holds that proprioception is task-dependent (46); by testing within the specific dynamic action of landing, it gives a truer measure of the sensory deficits that actually contribute to functional instability (6). Therefore, landing proprioception should be prioritized as an essential metric in the clinical assessment of CAI. Future studies may further validate and extend this approach to other populations.

This study has two key limitations. First, all participants were recruited from a university and fell within the age range of 18–25, which restricts the generalizability of our findings to a wider age demographic. Second, the participants' sports specialization and competitive level were not analyzed, hindering assessment of its potential impact on proprioceptive tests.

## 5 Conclusion

Proprioceptive deficits in CAI are modality-specific, primarily affecting kinesthesia, force sense, and landing proprioception. Among the impaired sub-modalities, landing proprioception has the strongest predictive factor for CAI. Therefore, clinical assessment and rehabilitation should prioritize these sub-modalities and develop targeted rehabilitation protocols to prevent recurrent sprains.

## Data Availability

The original contributions presented in the study are included in the article/Supplementary Material, further inquiries can be directed to the corresponding author.
